# Assessing the Feasibility and Effects of Introducing the USA National Surgical Quality Improvement Program on Clinical Outcomes and Cost in Saudi Arabia: An Observational Study

**DOI:** 10.4103/JQSH.JQSH_1_20

**Published:** 2020-02-11

**Authors:** Shahenaz Najjar, Adel F. Almutairi, Rashad Massoud, Khaled Al-Surimi, Sami Boghdadly

**Affiliations:** 1 Department of Health Informatics, Arab American University, Ramallah, Palestine; 2 Department of Population Health, King Abdullah International Medical Research Center/King Saud bin Abdulaziz University for Health Sciences, Ministry of National Guard Health Affairs, Riyadh, Saudi Arabia; 3 Department of Science Technology, King Abdullah International Medical Research Center/King Saud bin Abdulaziz University for Health Sciences, Riyadh, Saudi Arabia; 4 University Research Co. (URC), Bethesda, MD, USA; 5 USAID Applying Science to Strengthen and Improve Systems Project (ASSIST), Chevy Chase, MD, USA; 6 Department of Health Systems and Quality Management, College of Public Health and Health Informatics, King Abdullah International Medical Research Center/King Saud bin Abdulaziz University for Health Sciences, Riyadh, Saudi Arabia; 7 Department of Primary Care and Public Health, School of Public Health, Imperial College London, London, UK; 8 Operation Room Services, King Abdulaziz Medical City, Ministry of National Guard Health Affairs, Riyadh, Saudi Arabia

**Keywords:** Adverse events, health and safety, quality in healthcare, Saudi Arabia, surgery

## Abstract

**Introduction:**

This study aimed at introducing a systematic clinical registry to assess the outcomes of surgical performances and the associated costs of surgical complications in hospitals of Saudi Arabia.

**Materials and Methods:**

This was an observational retrospective cohort study. Three large Saudi public hospitals from different regions participated in the study. A systematic sample consisting of 2077 medical records was retrospectively reviewed after being received from the hospitals' surgical wards. The inclusion criteria of the study were inpatients of the surgical cases, patients older than 18 years, and those who underwent major surgery under general anesthesia. The occurrence of adverse events in surgical wards and the direct costs associated with these surgical adverse events were estimated. Results were reported in terms of odds ratio and 95% confidence interval. A value of *p* < 0.05 was considered statistically significant.

**Results:**

Introducing the systematic clinical registry to assess surgical outcomes and complications across multiple hospital sites is feasible. The findings of the study suggest that some areas are exemplary and others need improvement, such as sepsis cases, renal failure, ventilator use for more than 48 h, urinary tract infection, surgical site infection (SSI), length of stay after colorectal surgery, and rehospitalization. Additional costs from surgical complications in Riyadh only were approximately 0.5 million Saudi Arabian Riyal (127,764.40 USD) during that year. Most of the additional costs were due to sepsis and SSI.

**Conclusion:**

Empirical evidence derived from the idea of introducing a National Surgical Quality Improvement Program might be generally applicable to other countries in the region and worldwide, and can be used to measure surgical adverse events and track interventions over time. As a result, quality improvement initiatives could be identified to be implemented immediately focusing on preventing several surgical adverse events. A future study is needed to explore the underlying factors that contribute to the occurrence of surgical adverse events to be prevented and/or mitigated.

## Introduction

Recently, healthcare systems worldwide have undergone major transformations, including many quality improvement initiatives that aim at improving patients' safety from avoidable harm and ultimately improving the quality of healthcare services for patients and their families. These initiatives have contributed to reducing organizational healthcare costs as well as the burden of personal costs for those affected, including patients and healthcare workers. On the contrary, poor-quality healthcare always results in unnecessary spending, such as overuse of tests and procedures, treatment of hospital-acquired infections, and complications caused by bad management of chronic diseases.[1–4] Likewise, surgical complications are associated with increased length of hospital stay, cost of treatment, morbidity, and rehospitalization, which is a frequent, costly, and sometimes a life-threatening event that is associated with gaps in follow-up care. Surgical complications are often presented as a measure of the clinical performance of hospitals. In 2008, the annual direct medical costs resulting from adverse events in all settings, including surgical interventions, in the USA were estimated to be $19.5 billion.[5] In the United Kingdom, the cost of preventable adverse events incurred ranged from £1 to £2.5 billion,[6] and it was estimated to be $1.2 billion annually in Australia.[7]

Evaluating clinical outcomes and comparing all healthcare facilities to best standard benchmarks are crucial for ensuring the quality and safety of health services. Although measuring clinical outcomes across all specialties is of paramount importance, it is particularly vital to determine surgical outcomes, for which several mechanisms and programs can be used.[8] Most quality programs and initiatives use the easily obtainable claims or billing data. However, the claims data are limited, inconsistent, and subject to interpretation when used to measure quality and value of care. In addition, most of these measures are selective and determined by healthcare facilities.[9] Moreover, such evaluations do not take into account the stratification of risks, have a benchmark, or include proper patient follow-up mechanisms. On the contrary, the American College of Surgeons National Surgical Quality Improvement Program (ACS NSQIP) has developed a quality improvement initiative program that is widely used to improve surgical care. It was developed to determine patients' preoperative risk factors and postoperative outcomes, as well as to provide analytical tools for proper risk adjustment.[10] A study was conducted in the USA over 4 years to determine the validity of the risk-adjusted surgical mortality and morbidity rates model called NSQIP. The results of the NSQIP were impressive: there was approximately 27% decrease in 30-day surgically associated mortality and approximately 45% decrease in 30-day surgically associated morbidity.[11]

Another study was conducted to compare ACS NSQIP data with administrative and claims data collected by the University Health System Consortium (UHC) program.[12] It showed that the ACS NSQIP identified 28% more complications than the UHC, including 13% more surgical site infections (SSIs). Such findings revealed the usefulness and effectiveness of such a program in improving the quality and safety of care in surgical wards. Hence, it became the leading national risk-adjusted, outcome-based program in the USA, Canada, some Arab countries, and others.[13–15]

In the Kingdom of Saudi Arabia (KSA), healthcare services are provided mainly by public hospitals, including the Ministry of Health's hospitals and military hospitals. The latter refers to the Ministry of National Guard Health Affairs and the Ministry of Defense and Aviation. During the last few decades, these hospitals have introduced and implemented several quality improvement initiatives, particularly in surgical wards. Despite the benefits associated with these initiatives, the administrative data were not comprehensive. The hospitals had trouble tracking surgical complications and lacked the requisite data for analyses, which were important for assessing and planning quality of care improvements as well as for maintaining patient safety and developing positive interventions.

Therefore, this study aimed at testing the feasibility of adopting the NSQIP as an evidence-based tool to improve the quality of care for surgical patients in the KSA, starting at King Abdulaziz Medical Cities covering three regions in Saudi Arabia: central, eastern, and western. This decision was made based on the impressive results of the NSQIP reported in US hospitals, including (1) rapid decrease in postoperative mortality, (2) lower complication rates, (3) reduced care disparities, and (4) decreased spending.[16,17] This program differs from other quality improvement initiatives because it was designed to collect unbiased data, which allows for in-depth and meaningful analyses. We hope that the findings of this study will provide local evidence on how to develop nationwide improvement initiatives to improve clinical outcomes and associated costs.

## Materials and Methods

This was a cohort retrospective review based on surgical records for patients who underwent major surgeries under general anesthesia, deploying the idea of ACS NSQIP. We reviewed clinical data and outcomes of 30 days after surgery applying highly standardized and validated tool to collect clinical data. Several variables were collected, including patient demographics, surgical profile, preoperative risk assessment (such as pulmonary, cardiac, renal, nutritional, and immune measures), preoperative laboratory test results, operative information, postoperative occurrences within 30 days (including those involving the wound, urinary tract, or cardiac system), and postoperative information (such as hospital discharge, readmission, mortality, and reoperation).

The inclusion criteria of the study were inpatients who were admitted in surgical wards from July 1, 2015 till June 30, 2016. The exclusion criteria of the study were inpatients who were younger than 18 years; patients who had transplantation, trauma, hyperthermic intraperitoneal chemotherapy; and patients with more than three inguinal herniorrhaphies, breast lumpectomies, laparoscopic cholecystectomies, transurethral resection of bladder tumors, or bladder tumor cystoscopy resections in an 8-day period. The data were extracted from the ACS NSQIP database, a clinical registry to assess surgical complications and identify areas for improvement. This review was conducted in three regional hospitals in KSA: central, western, and eastern. The three participating hospitals are accredited by the Joint Commission International; the total number of beds was 1500 in the central region (CR), 751 in the western region (WR), and 400 in the eastern region (ER). The number of annually reported surgeries from July 1, 2015 to June 30, 2016 was 9561 in the CR, 7569 in the WR, and 3667 in the ER.

To avoid selection bias, a systematic sampling process was used for the selection of cases from operation logs, using an 8-day-cycle schedule with 42 obligatory cycles generated per year from July 1, 2015 to June 30, 2016. From each cycle, 40 cases were selected for review. To maintain data uniformity and reliability, the tool included standardized definitions and procedures. Moreover, initial online training for the reviewers, regular surgical clinical reviewer and surgeon champion conference calls, inter-rater reliability audits, and international conferences for all ACS NSQIP surgical clinical reviewers and surgeon champions were used to ensure reliability. Before conducting the main fieldwork, a pilot study was conducted on 20% of the selected patient records per year to assess the inter-rater reliability audit.

The review included the following diverse cases: general, vascular, and subspecialty surgical cases (neurology, orthopedics, plastic, thoracic, gynecology, cardiac, pediatric, and ophthalmology) as well as specific surgical cases, such as total knee arthroplasty and colectomy. After the medical record numbers were selected, the electronic medical records were retrieved and reviewed to collect preoperative risk factors, demographic data, preoperative laboratory test results, intraoperative data, clinical variables and complications, postoperative data, and 30-day outcomes (inpatient and outpatient). Then, 30 days after the surgery, the cases were reviewed again for postoperative outcome information (morbidity and mortality). The data were then entered into the ACS NSQIP database. Within the various surgical settings, the following adverse events were analyzed: mortality, morbidity, cardiac, pneumonia, unplanned intubation, ventilator use >48 h, venous thromboembolism, renal failure, urinary tract infection (UTI), SSI, sepsis, *Clostridium difficile* colitis, patient's out of room time, time patient left the operating room/postanesthetic room, and readmission. This study was approved by the institutional review board office at King Abdullah International Medical Research Center, Saudi Arabia (research protocol no. RC17/309/R).

### Review process, ACS NSQIP database, and data analysis

In each participating hospital, an independent trained surgical clinical reviewer was assigned to collect postoperative data for up to 30 days for each case. Moreover, a surgeon champion was assigned to lead and direct NSQIP implementation and improvement initiatives at each hospital. Data from each hospital were transferred into the ACS NSQIP headquarters in the USA. During the said period, 664 hospitals participated in this program worldwide; of which, 587 were from the USA and 61 from Canada. The ACS NSQIP headquarter team benchmarked and analyzed the risk adjustment (on specific measures), case-mix-adjusted, and postoperative 30-day morbidity and mortality of the participated international hospitals. To facilitate the benchmarking, several variables will be taken into consideration, including the number of beds as well as academic verses nonacademic and specialized hospitals. Risk adjustment is a statistical method used to fairly compare hospitals by correcting for differences in patient types and performance of higher-risk procedures. Moreover, and as hospitals used different patient characteristics to determine which cases underwent various surgeries, hierarchical statistical modeling was used to calculate the quality metrics of these hospitals. Those metrics were reported as hospital odds ratios. The 95% confidence interval was calculated for each odds ratio. According to the ACS NSQIP manual for 2016, an odds ratio equal to 1.0 meant that the hospital was performing as expected, whereas an odds ratio greater than 1.0 meant that the hospital was performing poorer than expected, and an odds ratio less than 1.0 meant that the hospital was performing better than expected. Thus, the results of the participating hospitals were rated as “exemplary,” “as expected,” or “needs improvement.” A hospital was categorized as needing improvement when it was determined to be either a “high outlier” or in the “10th decile.” In addition to identifying areas for improvement across the participated hospitals, we estimated the additional direct cost due to adverse events and surgical complications in the hospital at the CR. The measured costs did not include the costs associated with increased social care needs and lost productivity in time off work because we were unable to estimate these costs. Therefore, to estimate the direct costs, all cases identified with additional length of stay than recommended from evidence based were calculated. Then, we multiplied this number of cases with the additional costs per case. The additional costs per case were estimated based on literature documentation.[18–21]

All statistical analyses were conducted using SAS System for Windows version 9.2 (SAS Institute, Cary, North Carolina).

## Results

### Patient characteristics

**Figure 1:**
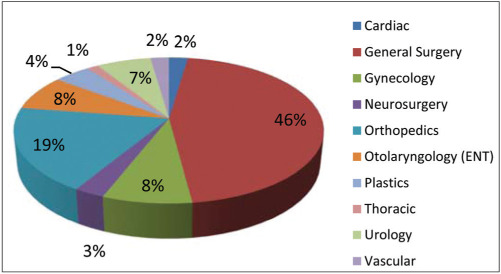
Distribution of surgery type. ENT = ear, nose, and throat

In total, 2077 cases were reviewed from the three regions: 847 (41%) cases from the CR, 278 (13%) from the WR, and 952 (46%) from the ER. A total of 55% of the participants were women. The mean age of the participants was 46 years (standard deviation [SD] = 16.97; range, 18.0–102.0 years). The mean length of stay was 7 days (SD = 15.86; range, 0–34 days). The health status of 89% of the patients was considered independent. Approximately 45% of the operations were general surgery. The distribution of the operations conducted by surgical specialties is shown in Figure 1.

### Clinical performance outcomes and areas for improvement

**Figure 2:**
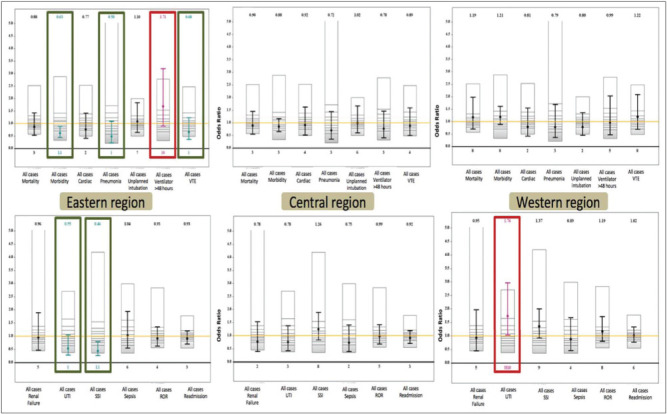
Exemplary and need improvement areas. VTE = venous thromboembolism, UTI = urinary tract infection, SSI = surgical site infection, ROR = return to operating room

The clinical performance of the ER was rated “exemplary” in morbidity, pneumonia, UTI, and SSI. However, ventilator use >48 h was identified as an area that needed improvement. The CR was rated “as expected” in all general cases of complications. The WR was rated “as expected” in all cases of complications and “needed improvement” in UTI [Figure 2].

Regarding surgical specialties, the review revealed that the ER was a statistical outlier and needed improvement in the following areas: sepsis, renal failure, ventilator use >48 h, and readmission. The areas that needed improvements in the CR had a higher risk of sepsis, SSI, return to the operating room, readmission, aortoiliac (blockage of the aorta), targeted orthopedic total knee arthroplasty morbidity, and colorectal surgery length of stay. The areas that needed improvements are presented in Tables 1–3.

**Table 1:** Complication scores stratified by specialty: eastern region

**Surgical specialty**	**Type of complication**	**Odds ratio**	**Lower CI**	**Upper CI**	**Score**
General/Vascular	Pneumonia	0.59	0.27	1.32	Exemplary
	SSI	0.63	0.37	1.08	Exemplary
	**Sepsis**	**1.79**	**0.95**	**3.35**	**Needs improvement**
	**Renal failure**	**1.42**	**0.76**	**2.66**	**Needs improvement**
	Morbidity	0.68	0.46	1.01	Exemplary
	Pneumonia	0.62	0.27	1.40	Exemplary
	SSI	0.60	0.35	1.04	Exemplary
Vascular	**Ventilator >48 h**	**1.36**	**0.59**	**3.11**	**Needs improvement**
	**Renal failure**	**1.37**	**0.60**	**3.16**	**Needs improvement**
	**Sepsis**	**1.39**	**0.58**	**3.35**	**Needs improvement**
Subspecialties	**Neurosurgery ventilator**	**1.60**	**0.56**	**4.61**	**Needs improvement**
	**>48 h**				
	Orthopedic morbidity	0.67	0.39	1.16	Exemplary
	Orthopedic VTE	0.74	0.34	1.59	Exemplary
Measure DSM		0.76	0.57	1.00	Exemplary
**T GEN appendectomy readmission**		**1.40**	**0.76**	**2.55**	**Needs improvement**
**T NSG spine ventilator >48 h**		**1.39**	**0.42**	**4.66**	**Needs improvement**

Bold face indicates statistical significance (*p* < 0.05). CI = confidence interval, SSI = surgical site infection, VTE = venous thromboembolism, DSM = death or serious morbidity, T GEN = targeted general, T NSG = targeted neurosurgery

The high risk of surgical complications in the CR necessitates calculation of the costs for each surgical complication to project the magnitude of the issue that drains the organization's financial resources.

### Estimated associated costs

The total additional cost from surgical complications in Riyadh only was approximately 0.5 million Saudi Arabian Riyal (127,764.40 USD) during that year. Most of the additional costs were due to sepsis (66,808 USD) and SSI (43,169.74 USD). Table 4 shows in detail the expected cost associated with each additional complication.

**Table 2:** Complication scores stratified by specialty: central region

**Surgical specialty**	**Type of complication**	**Odds ratio**	**Lower CI**	**Upper CI**	**Score**
Colorectal	**Colorectal length of stay**	**1.74**	**0.77**	**3.91**	**Needs improvement**
Vascular	**Sepsis**	**1.33**	**0.57**	**3.13**	**Needs improvement**
Subspecialties	**Cardiac SSI**	**1.21**	**0.52**	**2.80**	**Needs improvement**
	**Orthopedic SSI**	**1.60**	**0.71**	**3.60**	**Needs improvement**
	**Otolaryngology ROR**	**1.21**	**0.62**	**2.37**	**Needs improvement**
	**Otolaryngology readmission**	**1.12**	**0.70**	**1.79**	**Needs improvement**
	Plastic SSI	0.76	0.30	1.93	Exemplary
Length of stay for:	**Colectomy**	**1.71**	**0.77**	**3.78**	**Needs improvement**
	**VASC aortoiliac (open)**	**1.47**	**0.52**	**4.20**	**Needs improvement**
	**VASC lower extremity (open)**	**1.93**	**0.60**	**6.24**	**Needs improvement**
**T GEN appendectomy SSI**		**1.47**	**0.54**	**3.99**	**Needs improvement**
**Targeted ORTHO TKA morbidity**		**1.44**	**0.70**	**2.93**	**Needs improvement**
**Targeted ORTHO TKA SSI**		**1.86**	**0.57**	**6.01**	**Needs improvement**
**Targeted ORTHO Hip fracture SSI**		**1.59**	**0.39**	**6.49**	**Needs improvement**
Targeted plastic abdominoplasty SSI		0.69	0.09	5.16	Exemplary

Bold face indicates statistical significance (*p* < 0.05). CI = confidence interval, SSI = surgical site infection, VTE = venous thromboembolism, ROR = return to operating room, VASC = vascular surgery case, T GEN = targeted general, ORTHO = orthopedics, TKA = total knee arthroplasty

**Table 3:** Complication scores stratified by specialty: western region

**Surgical specialty**	**Type of complication**	**Odds ratio**	**Lower CI**	**Upper CI**	**Score**
Colorectal	**Colorectal length of stay**	**1.65**	**0.81**	**3.38**	**Needs improvement**
Subspecialties	**Orthopedic UTI**	**1.95**	**0.81**	**4.70**	**Needs improvement**
	**Thoracic mortality**	**1.18**	**0.50**	**2.78**	**Needs improvement**
	**Urology morbidity**	**1.31**	**0.68**	**2.54**	**Needs improvement**
	Gynecology morbidity	0.73	0.38	1.41	Exemplary
	Plastic morbidity	1.21	0.62	2.37	Exemplary
	Plastic SSI	1.12	0.70	1.79	Exemplary
	Plastic ROR	0.77	0.36	1.64	Exemplary
	Plastic readmission	0.75	0.33	1.73	Exemplary
**Measure UTI**		**1.96**	**1.06**	**3.64**	**Needs improvement**
**Emergency T GEN mortality**		**1.20**	**0.59**	**2.46**	**Needs improvement**

Bold face indicates statistical significance (*p* < 0.05). CI = confidence interval, UTI = urinary tract infection, SSI = surgical site infection, ROR = return to operating room, T GEN = targeted general

**Table 4:** Additional costs for each surgical complication in central region

**Surgical complications**	**Subspecialty**	**Additional number of events**	**Additional cost/complication (USD)**	**Total estimation (USD)**
Length of stay (LOS)	Colorectal/colectomy	3	5,597.98	8,190.12
	Vascular aortoiliac (open)	2	1,296.07	
	Vascular lower extremity (open)	2	1,296.07	
Sepsis	Sepsis in vascular	2	66,808	66,808
Surgical site infection (SSI)	Cardiac	1	20,556.71	43,169.74
	Orthopedic	2	8,530.26	
	Total knee arthroplasty Hip fracture	1	9,971.34	
	Appendectomy	2	41,114.27	
Return to operating room (ROR)	ROR in otolaryngology	2	9,596.55	9,596.55

USD = United States Dollar

The total additional costs from surgical complications in Riyadh = 127,764.40 USD

## Discussion

To the best of our knowledge, this was the first study to assess surgical complications in KSA using a systematic clinical database registry for general surgery. The results of the study indicated that using such a tool was feasible in three regional hospitals in KSA. Some of the general surgery outcomes received excellent scores as compared to other NSQIP users as an external benchmark. We defined the procedures and areas that were rated “needs improvement” within the database and provided suggestions to improve patient outcomes. The areas that were rated as “needs improvement” differed in the three regions. The results showed that the expected cost associated with additional complications was approximately half million in Riyadh only. This calculation projected the magnitude of the issue that drained the organization's financial resources due to surgical complications.

Sepsis was presented as an area that needed improvement in the CR and the ER. One of nine (11.76%) and one of 12 (8.33%) vascular patients in the CR and ER, respectively, had sepsis. The observed rates were higher than the expected rates (2.84% and 1.24%, respectively) in both sites. Sepsis is a “time-critical condition that can lead to organ damage, multi-organ failure, septic shock, and eventually death.” It has major impacts on healthcare resources and expenditures.[22,23] The estimation of sepsis incidence varies across countries; however, it is approximately 300 cases per 100,000 persons per year.[22] Evidences indicate that early and appropriate therapy for patients with sepsis improves the outcomes.[24,25] Gatewood *et al*.[26] suggested a three-tiered protocol as an intervention method to improve sepsis outcomes. Their protocol consists of “(1) a nurse-driven screening tool and management protocol to identify and initiate early treatment of patients with sepsis, (2) a computer-assisted screening algorithm that generated a ‘Sepsis Alert’ pop-up screen in the electronic medical record for treating clinical healthcare providers, and (3) automated suggested sepsis-specific order sets for initial workup and resuscitation, antibiotic selection, and goal-directed therapy.”[26] There is a need to analyze the root causes of sepsis in CR and ER hospitals and then follow appropriate protocols and standards to improve sepsis outcomes.

Another area identified as “needs improvement” was for patients on ventilator for more than 48 h. The long-term ventilation problem was observed in vascular and neurosurgery spine operations. It has been observed that approximately 9%–27% of patients who were put on ventilator for more than 48 h develop ventilator-associated pneumonia (VAP) during their stay in the intensive care unit at the hospital.[27,28] As a result, the hospital length of stay is increased by 7–9 days, with crude mortality rates as high as 70%, although the mortality due to VAP has been estimated to be between 33% and 50%.[24,25,29] VAP also leads to a significant financial burden on the healthcare system.[25,30–32] Adherence to evidence-based protocols for ventilation will reduce the patient's significant physical and financial risks in the ER hospital.

SSIs were defined as adverse events in cardiac, orthopedic, and appendectomy subspecialty surgeries in CR. Comparing these results with the expected rate worldwide, an alarming SSI rate was found. SSI is the second leading cause of the nosocomial infection worldwide. It is a major source of postoperative morbidity and mortality and represents a major financial burden to the healthcare system, with an estimated direct cost of $3.45–10.07 billion in 2007.[33] Active surveillance, risk assessment, and following evidence-based guidelines for infection control will help reduce SSI rates and increase quality improvement. Moreover, working on the modifiable process-related (exogenous) variables of SSI (such as nutritional statuses, tobacco use, correct use of antibiotics, and the intraoperative technique) will be beneficial in reducing this event.

In addition, the results of this study showed that renal failure is an area that needs improvement (8.90%) in vascular surgery in ER hospital. Postoperative renal failure is a leading cause of morbidity, mortality, and prolonged hospitalization, resulting in increased hospital costs. Many factors can contribute to postoperative renal failure. Therefore, in-depth analyses should be conducted at each facility to explore risk factors associated with this postoperative complication. Moreover, further analysis and root cause analysis tools should be conducted to determine the reasons behind otolaryngology readmission and recommend approaches to reduce the length of stay following colorectal surgery, vascular surgery case (VASC) aortoiliac open, and VASC lower extremity open. To improve the length of stay, different approaches can be used, such as enhanced recovery after surgery (ERAS) approach. ERAS is a multimodal approach to perioperative care that combines a range of interventions to enable early mobilization and feeding after surgery.[34]

### Future implications

Overall, the results of this study show the feasibility of using a systematic clinical registry to assess surgical complications and identify areas of improvement in hospitals across the KSA. However, further investigation must be conducted to identify risk factors associated with each surgical complication and to study potential confounders that may impact the results of specific research study questions. Specific clinical research questions that best fit the available variables in the NSQIP dataset can be formulated. Identifying risk factors for adverse events is critical for establishing quality improvement protocols. NSQIP provides a lot of information that can be used to prioritize quality improvement efforts for general surgery subspecialties for patient populations. Although members of each surgery subspecialty should engage in actions that specifically provide quality improvement in their designated area, multidisciplinary collaboration among hospital administrations, quality management, and clinical healthcare providers from all specialties will be needed to promote success. The future implemented interventions should be system-approach interventions that manage the processes of care, not the physicians and nurses only.

### Limitations

This study has several limitations that should be acknowledged. First, our study applied a retrospective method and was unable to identify all potential confounders. However, the use of the NSQIP provided a general overview of the areas that needed improvement. Further analysis will be needed to identify confounders. The mortality data were based on all-cause mortality. The tool does not allow for additional details on the cause of death, potentially limiting the interpretation of mortality data. Moreover, the NSQIP tool uses Current Procedural Terminology codes, whereas the electronic medical records at the hospitals use *International Classification of Diseases*, *Tenth Revision*, *Clinical Modification* (*ICD-10-CM*) codes. This necessitates the recording of needed variables before submission to the NSQIP database. Using *ICD-10-CM* coding will facilitate the transfer of data to the NSQIP database. The implementation of NSQIP program faced an under-recruitment problem in WR. Shortage in the reviewers' team might be the reason behind the under-recruitment. Providing enough team members to review the patients' records in WR is needed to be able to implement the program efficiently and effectively.

## Conclusion

The success derived from this study may be generally applicable to other countries in the region. To avoid additional personal and institutional costs, interventions should be implemented immediately. Quality improvement efforts should focus on sepsis, SSI, ventilator use for more than 48 h, and length of stay. Future investigation could reveal the underlying causes that contribute to these surgical complications, allowing for the implementation of appropriate interventions and measurement of their effectiveness in patient safety and quality healthcare improvement. A multidisciplinary team should work together in a structured and well-organized manner to improve the quality of these hospitals.
